# Endoscopic ultrasound-guided enteroenterostomy with lumen-apposing metal stent for post-gastrectomy afferent loop obstruction

**DOI:** 10.1055/a-2629-6766

**Published:** 2025-07-15

**Authors:** Mengmeng Zhang, Yunlu Feng, Wen Shi, Aiming Yang

**Affiliations:** 1 34732 Department of Gastroenterology, Peking Union Medical College Hospital, Beijing, China

Afferent loop obstruction is a complication following upper gastrointestinal bypass surgeries 1 that can precipitate pancreatitis, cholangitis, or perforation, etc. Timely interventions are therefore required to avoid severe complications.

A 61-year-old man who underwent a total gastrectomy and esophagojejunostomy (Roux-en-Y) for gastric signet ring cell carcinoma three years previously was admitted to our hospital with abdominal pain and vomiting. Computed tomography ( Fig. 1 ) revealed dilation and gas-liquid levels of the afferent loop without signs of cancer recurrence. The gastroscope and guidewire were unable to proceed deeply to the afferent loop through the end-to-side jejuno-jejunal anastomosis because of significant angulation. Therefore, we attempted endoscopic ultrasound (EUS)-guided enteroenterostomy using a lumen-apposing metal stent (LAMS) to relieve afferent loop obstruction Video 1 , Fig. 2 ). A guidewire was placed into the efferent loop under gastroscopy. A linear echoendoscope (GF-UCT180; Olympus Medical Systems, Tokyo, Japan) was delivered to the efferent loop assisted with an endoloop along the guidewire, and the dilated afferent loop was displayed on the EUS image. A 15-mm LAMS (Hot AXIOS; Boston Scientific Corp., Marlborough, Massachusetts, USA) was deployed across the afferent and efferent loops. A large amount of intestinal fluid was extruded into the efferent lumen via the LAMS. The clinical complaints were resolved, and after three days, oral intake was recovered without vomiting or pain. After one week, CT ( Fig. 3 ) showed improvement in the dilation of the afferent loop. No complications were seen during the follow-up.

**Fig. 1:**
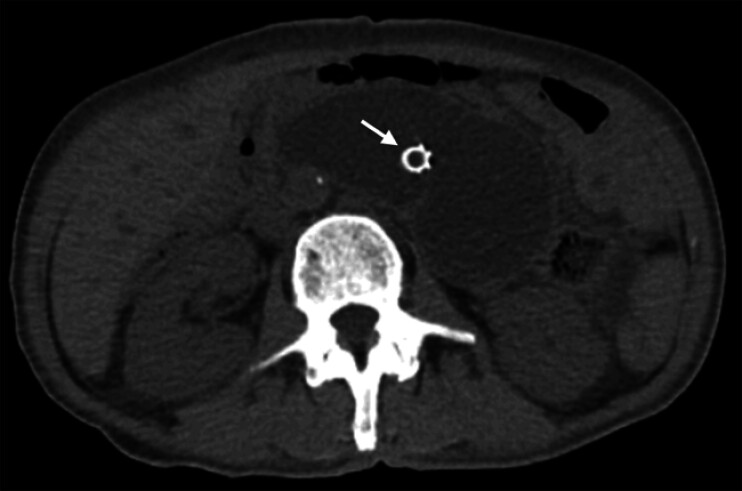
Preprocedural computed tomography (CT) scan imaging. Preprocedural CT revealed the dilation of the afferent loop in a 61-year-old man who had undergone total gastrectomy and esophagojejunostomy (Roux-en-Y) for gastric signet ring cell carcinoma three years previously. The patient had a biliary stent for obstructive jaundice (white arrow).

Endoscopic ultrasound-guided enteroenterostomy with lumen-apposing metal stent for post-gastrectomy afferent loop syndrome.Video 1

**Fig. 2:**
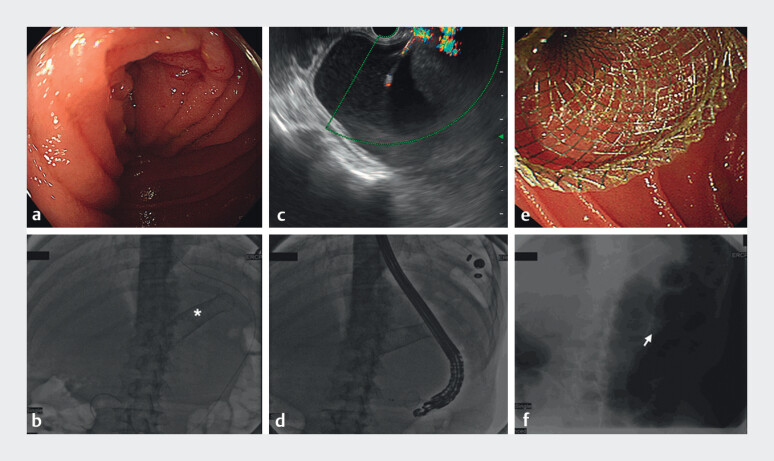
Endoscopic ultrasound-guided enteroenterostomy with a lumen-apposing metal stent (LAMS). **a** Gastroscopy revealed a tight luminal narrowing without definitive abnormal mucosal lesions in the afferent loop. **b** A guidewire was passed into the efferent lumen under gastroscopy (white star represents colonic stent for colonic obstruction previously). **c** Under endoscopic ultrasound, the delivery catheter was introduced into the afferent loop through the guidewire after the afferent loop was punctured using the electrocautery-enhanced LAMS connected to an electrosurgical unit. **d** The LAMS was deployed along the guidewire with fluoroscopic guidance. **e** Final gastroscopic view of the LAMS in the efferent lumen. **f** X-ray radiography showed the LAMS across the afferent and efferent loops (white arrow).

**Fig. 3:**
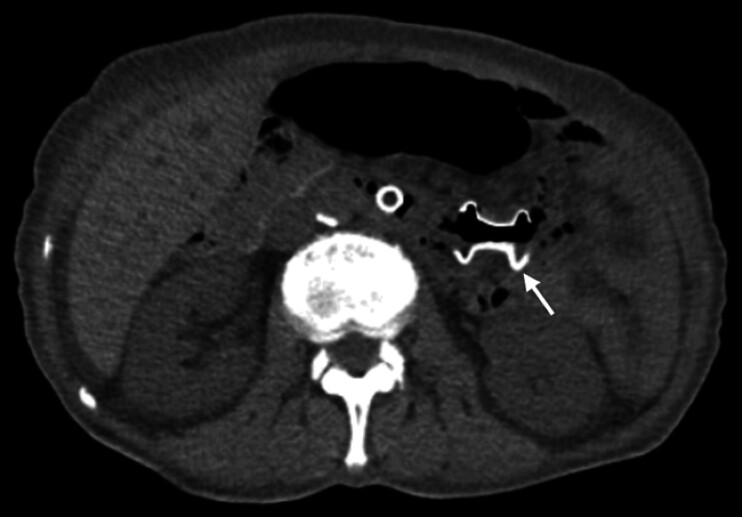
Postprocedural computed tomography (CT) scan imaging. CT on postoperative day 7 revealed the improvement in the dilation of the afferent loop, and the LAMS (white arrow) was deployed across the afferent and efferent loops.

Therefore, EUS-guided enteroenterostomy with a LAMS is a technically feasible, effective, and minimally invasive procedure for afferent loop obstruction. Notably, EUS-guided gastroenterostomy is appropriate for surgical patients with a remnant stomach 1 2 3 , whereas enteroenterostomy or external drainage are optional measures for patients undergoing total gastrectomy. Due to the advantages of no enteral fluid loss and better quality of life, EUS-guided enteroenterostomy should be the prioritized therapeutic approach for afferent loop obstruction in patients undergoing total gastrectomy.

Endoscopy_UCTN_Code_TTT_1AS_2AG

## Notes

Endoscopy E-Videos https://eref.thieme.de/e-videos
*E-Videos* is an open access online section of the journal *Endoscopy* , reporting on interesting cases and new techniques in gastroenterological endoscopy. All papers include a high-quality video and are published with a Creative Commons CC-BY license. Endoscopy E-Videos qualify for HINARI discounts and waivers and eligibility is automatically checked during the submission process. We grant 100% waivers to articles whose corresponding authors are based in Group A countries and 50% waivers to those who are based in Group B countries as classified by Research4Life (see: https://www.research4life.org/access/eligibility/ ). This section has its own submission website at https://mc.manuscriptcentral.com/e-videos .
